# Feast of Sacrifice and Orf, Milan, Italy, 2015–2018

**DOI:** 10.3201/eid2508.181063

**Published:** 2019-08

**Authors:** Stefano Veraldi, Luigi Esposito, Paolo Pontini, Fabrizio Vaira, Gianluca Nazzaro

**Affiliations:** Università degli Studi di Milano, Milan, Italy

**Keywords:** Orf virus, ecthyma contagiosum, skin infection, Parapoxvirus, epidemiology, Italy, viruses, zoonoses

## Abstract

Orf (ecthyma contagiosum) is an infection of the skin caused by a DNA virus belonging to the genus *Parapoxvirus*. We recently observed 7 cases of orf in Muslim men living in the metropolitan area of Milan, Italy, who acquired the infection after the Feast of Sacrifice.

Orf (ecthyma contagiosum) is an infection of the skin caused by a DNA virus of the genus *Parapoxvirus*, family *Poxviridae*. Skin lesions (e.g., vesicles, blisters, pustules, erosions, ulcers, papules, nodules) occur at sites of inoculation of the virus 3–15 days after infection. Hands are usually affected (*1*). The differential diagnosis for orf includes milker’s nodule, anthrax, tularemia, fish tank granuloma, cutaneous leishmaniasis, pyogenic granuloma, and keratoacanthoma (*1*). The disease spontaneously heals within 6 weeks, although pain, bacterial superinfections, and regional lymphadenitis are possible (*1*). Treatment is based on topical antiseptics (*1*).

Orf virus usually infects sheep and goats. Humans are infected by handling infected meat from these animals; orf is considered an occupational disease in shepherds, shearers, veterinarians, butchers, and cooks (*1*). In the last few years, orf has occurred after the Muslim Feast of Sacrifice (Eid al-Adha) (*1*–*10*). In 2014, we reported a case of orf that appeared after sheep slaughtering for this feast (*1*). During 2015–2018, we observed 7 additional cases in Muslim men 18–61 years of age who were of Moroccan, Tunisian, or Egyptian origin. They had been infected 2–3 weeks after lamb slaughtering for the feast (Table). One patient was a butcher. In all patients, 1 hand and/or fingers were involved. In 4 patients, orf presented with erythematous pustules; 3 of these patients had ulcerated nodules. In all patients, clinical diagnosis was confirmed by histopathologic examinations.

**Table:** Characteristics of orf in Muslim men after Feast of Sacrifice, Milan, Italy, 2015–2018

Patient no.	Age, y	Location	Characteristic
1	34	Left hand	Erythematous, ulcerated nodule
2	42	Back of right hand	Erythematous pustule
3	44	Back of right hand	Erythematous pustule
4	18	Back of second right finger	Ulcerated nodule
5	21	Palm of right hand	3 Erythematous pustules
6	44	Back of second left finger	Erythematous pustule
7	61	Third right finger	Ulcerated nodule

Orf acquired during the Feast of Sacrifice was reported in 1982 in Turkey (*2*). Other cases were subsequently reported in France (*3*,*10*), Belgium (*4*,*5*), Italy (*1*), Turkey (*6*,*7*,*9*), and the United States (*8*). Epidemics also were reported: in Belgium, 23 cases in 2000 and 44 cases in 2001 (*4*,*5*), and in Turkey, 9 cases in 2005 and 29 cases in 2009 (*6*,*7*).

In most reported cases, orf appeared days or weeks after the Feast of Sacrifice. This feast is celebrated 2 months and 10 days after the end of Ramadan; the exact date varies (*4*). During the feast, many Muslim families kill a lamb, which has to be bled alive (*4*). Only men may kill the lamb. Orf occurs more often in men; however, it occurs also in women, who often handle the infected meat with bare hands during preparation and cooking.

In conclusion, in the metropolitan area of Milan, where ≈250,000 Muslims reside, we recently observed 7 patients with orf acquired during the Feast of Sacrifice. Prevention measures are difficult. For example, most of the Muslims living in the metropolitan area of Milan are Moroccans, who travel to Italy by car, carrying the infected meat; thus, no prevention measures have been taken.
